# Influencing Cancer Screening Participation Rates—Providing a Combined Cancer Screening Program (a ‘One Stop’ Shop) Could Be a Potential Answer

**DOI:** 10.3389/fonc.2017.00308

**Published:** 2017-12-13

**Authors:** Amanda Bobridge, Kay Price, Tiffany K. Gill, Anne W. Taylor

**Affiliations:** ^1^University of South Australia, Adelaide, SA, Australia; ^2^The University of Adelaide, Adelaide, SA, Australia

**Keywords:** cancer screening, combined screening, screening behaviors, combined cancer screening, screening participation

## Abstract

**Introduction:**

Participation in established cancer screening programs remains variable. Therefore, a renewed focus on how to increase screening uptake, including addressing structural barriers such as time, travel, and cost is needed. One approach could be the provision of combined cancer screening, where multiple screening tests are provided at the same time and location (essentially a ‘One Stop’ screening shop). This cohort study explored both cancer screening behavior and the acceptability of a combined screening approach.

**Methods:**

Participants of the North Western Adelaide Health Study (NWAHS), South Australia were invited to participate in a questionnaire about cancer screening behaviors and the acceptability of a proposed ‘One Stop’ cancer screening shop. Data were collected from 10th August 2015 to 18th January 2016, weighted for selection probability, age, and sex and analyzed using descriptive and multivariable logistic regression analysis.

**Results:**

1,562 people, 52% female (mean age 54.1 years ± 15.2) participated. Reported screening participation was low, the highest being for Pap Smear (34.4%). Common reasons for screening participation were preventing sickness (56.1%, CI 53.2–59.0%), maintaining health (51%, CI 48–53.9%), and free program provision (30.9%, CI 28.2–33.6%). Females were less likely to state that screening is not beneficial [OR 0.37 (CI 0.21–0.66), *p* < 0.001] and to cite sickness prevention [OR 2.10 (CI 1.46–3.00), *p* < 0.001] and free program [OR 1.75 (CI 1.22–2.51), *p* < 0.003] as reasons for screening participation. Of those who did not participate, 34.6% (CI 30.3–39.1%) stated that there was nothing that discouraged them from participation, with 55- to 64-year olds [OR 0.24 (CI 0.07–0.74), *p* < 0.04] being less likely to cite this reason. 21% (CI 17.2–24.8%) thought they did not need screening, while a smaller proportion stated not having time (6.9%, CI 4.9–9.7%) and the costs associated with screening (5.2%, CI 3.5–7.7%). The majority of participants (85.3%, CI 81.9–88.2%) supported multiple screening being offered at the same time and location.

**Conclusion:**

Identified screening behaviors in this study are similar to those reported in the literature. The high support for the concept of combined cancer screening demonstrates that this type of approach is acceptable to potential end users and warrants further investigation.

## Introduction

Despite cancer being one of the leading causes of morbidity and mortality (1), current cancer screening participation rates are variable, both internationally (2, 3) and in Australia (4). This variability is of concern, as it has been demonstrated that participating in organized cancer screening programs can identify cancer at an earlier stage, examples including bowel (5, 6), breast (7, 8), and cervical (9, 10), allowing for earlier detection and thus intervention in a person’s disease trajectory.

In the South Australian context, people currently have to schedule and find time to attend multiple appointments to achieve the recommended health and cancer screenings, with the clinics which offer these services being up to (depending upon location and mode of transport) 2 h away. In the broader, Australian context, in rural and remote regions, these services may not be able at all, necessitating long distance travel to the nearest biggest city to access the required services. These types of practical barriers to screening participation have also been well articulated in the literature, including time commitment, travel, administrative processes, and out-of-pocket costs (11–15). Given the aforementioned variability in screening participation, there is a need for a renewed focus on how these barriers can be circumvented to help improve cancer screening uptake.

Our team proposes that a potential solution to the problem of participation is the provision of a ‘One Stop’ combined health screening program, which, depending on age and gender would provide to people appropriate health and cancer screenings, inclusive of mammography, pap smear (women) prostate specific antigen screening (men), skin checks, and bowel cancer screening education at the same time and location. The overall objective of this program would be the earlier detection of factors that contribute to cancer and yet are amenable to intervention, such as smoking, alcohol consumption, and elevated body mass index (16) and identification of cancer at a potentially earlier stage.

However, before the implementation and evaluation of such a ‘One Stop’ program, a gauge of the acceptability to the end user at the community level must be established (17, 18). Therefore, the aim of this study was to investigate in an adult population, current cancer screening participation, reasons for non/participation, the support for a (theoretical) ‘One Stop’ screening shop (different types of cancer screening provide at the same time and location) and the factors that may influence these outcomes.

## Materials and Methods

This study involved participants of the ongoing North Western Adelaide Health Study (NWAHS) (19), which has been carried out in accordance with the recommendations of the National Statement on Ethical Conduct in Human Research (National Health and Research Council, Australia) with written informed consent from all subjects. All subjects gave written informed consent in accordance with the Declaration of Helsinki. The NWAHS protocol was approved by the Human Research Ethics Committees of the Queen Elizabeth and Lyell McEwin Hospitals (Adelaide, SA, Australia).

North Western Adelaide Health Study participants were invited to complete online or paper based questionnaires in relation to health issues and cancer screening, including; what type of screening the person was currently participating in, the benefit of screening, the enablers and barriers to screening participation and whether the participant would support the concept of different types of cancer screening being provided at the same time and location. If participants had not completed the questionnaire within 3 weeks, a reminder letter or e-mail was sent. Completion and return of the questionnaire was considered opting in and, therefore, consenting to study participation.

Data were collected from 10th August 2015 to 16th January 2016. In phase 1 (1999–2003) of the NWAHS, data were weighted by region (western and northern health regions), age group, sex, and probability of selection in the household to the Australian Bureau of Statistics 1999 Estimated Resident Population (20) and 2001 Census data (21). This weighting was undertaken to reflect the population of interest and to correct for potential non-response bias in which some groups of respondents may be over or underrepresented. For the current questionnaire study, data were weighted by sex, age group, and area of residence using 2011 Australian Census data (22) and incorporated the original weight from Stage 1 in the calculation.

Data were analyzed using IBM Statistics SPSS version 24 (IBM, Armonk, New York, NY, USA) and STATA version 13 (StataCorp, College Station, TX, USA), including descriptive analysis and multivariable logistic regression analysis, with age, sex, marital status, education, annual gross income, and family structure included as covariates within the model and non-significant variables (*p* > 0.05) removed in a backwards stepwise elimination.

## Results

**Figure 1:**
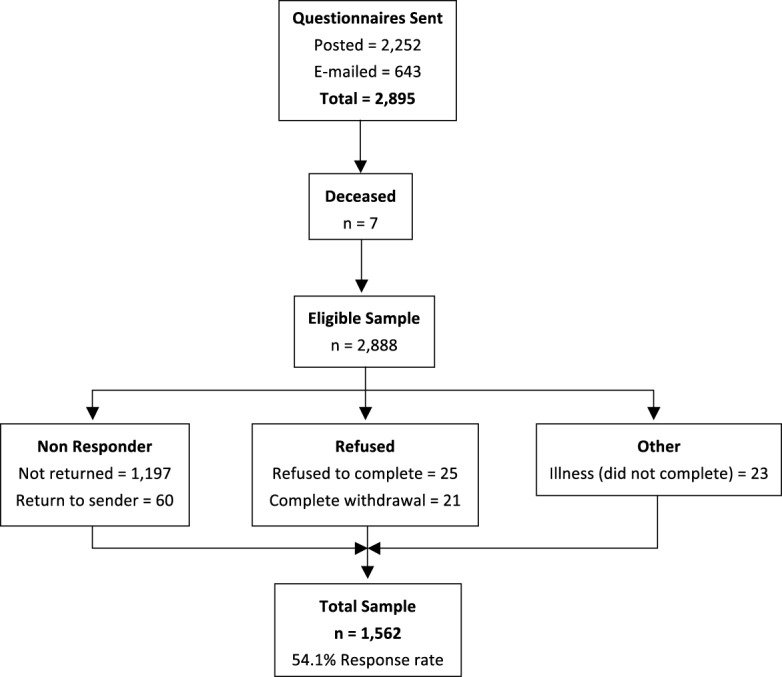
Study numbers: eligible, non-responders, refusal, other, and total.

The total number of questionnaires posted or e-mailed was 2,895, with 7 participants subsequently being identified as deceased, leaving an eligible sample of 2,888. The number of completed questionnaires returned was 1,562 (54.1% response rate, Figure 1). Fifty two percent of respondents were female and 48% were male, with a mean age of 54.1 years (±15.2) and the majority (77.2%) being of Australian birth origin. Table 1 presents the sociodemographic profile of the sample.

**Table 1:** Sociodemographic profile (*n* = 1,562).

Demographic	*n*	%	95% CI
**Age groups (years)**			
30–44	531	34	31.7–36.4
45–54	355	22.7	20.7–24.9
55–64	286	18.3	16.5–20.3
65–74	194	12.5	10.9–14.2
>75	194	12.4	10.9–14.2

**Country of birth**			
Australia	1,206	77.2	74.0–80.1
United Kingdom or Ireland	218	13.9	12.0–16.1
Europe, USSR, or Baltic States	83	5.3	3.8–7.4
Asia/other	54	3.5	2.1–5.7
Not stated	1	0.1	0.02–0.3

**Marital status**			
Married	977	62.5	60.1–64.9
Widowed	140	9	7.6–10.5
Divorced	114	7.3	6.1–8.7
Separated	94	6	5.0–7.3
Never married	148	9.5	8.1–11.0
Not stated	44	2.8	2.1–3.8

**Highest education**			
Some primary school	7	0.4	0.2–0.9
Complete primary school	34	2.2	1.6–3.0
Some high school	370	23.7	21.7–25.9
Complete high school	225	14.4	12.7–16.2
Tafe/apprenticeship	82	5.3	4.3–6.5
Trade cert or diploma	400	25.6	23.5–27.8
Bachelor or higher	395	25.3	23.2–27.5
Other	7	0.5	0.2–0.9
Not stated	43	2.7	2.0–3.7

**Annual gross income ($)**			
12,001–20,000	26	1.6	1.1–2.4
20,001–40,000	126	8	6.8–9.5
40,001–60,000	219	14	12.4–15.8
60,001–80,000	205	13.1	11.5–14.9
80,001–100,000	158	10.1	8.7–11.7
100,001–150,000	233	14.9	13.2–16.7
150,001–200,000	243	15.6	13.8–17.4
>200,000	104	6.7	5.5–8.0
Not sure	54	3.4	2.6–4.5
Not stated	114	7.3	6.1–8.7

**Family structure**			
Have children, living with both biological or adoptive parents	548	35.1	32.7–37.5
Step or blended family	65	4.2	3.3–5.3
Sole parent family	46	3	2.2–3.9
Shared care parenting	12	0.8	0.4–1.4
Adult living alone	249	15.9	14.2–17.8
Adult living with partner	422	27	24.9–29.3
Related adults living together	95	6.1	5.0–7.4
Related adults and children living together	19	1.2	0.8–1.9
Family/couples and unrelated adult/children living together	6	0.4	0.2–0.8
Unrelated adults living together	28	1.8	1.3–2.6
Other	12	0.7	0.4–1.3
Not stated	59	3.8	2.9–4.8

Despite the reported cancer screening participation rates being low (ranging from 3.5% for skin cancer screening up to 34.4% for Pap Smear; Table 2), 93.1% (CI 91.7–94.2%) of participants thought that screening for cancer was beneficial (Table 3). Multivariable regression analysis demonstrated that females [OR 0.37 (CI 0.21–0.66), *p* < 0.001] and adults living with a partner and no children [OR 0.42 (CI 0.20–0.91), *p* < 0.027] were less likely to state that screening is not beneficial.

**Table 2:** Proportion of respondents participating in cancer screening programs.

Type of screening	*n*	%	95% CI
PAP smear	538	34.4	32.1–36.8
Bowel cancer	532	34.1	31.7–36.4
Mammogram	449	28.7	26.5–31.0
Prostate cancer	272	17.4	15.6–19.4
Skin cancer	55	3.5	2.7–4.5
Other	14	0.9	0.5–1.5
None	445	28.5	26.3–30.8
Not stated	32	2	1.4–2.9

Total	1,562	100	

**Table 3:** Proportion of respondents who thought that screening for cancer is beneficial.

Response	*n*	%	95% CI
Yes	1,454	93.1	91.7–94.2
No	10	0.7	0.3–1.2
Not stated	11	0.7	0.4–1.3
Do not know	87	5.6	4.5–6.8

Total	1,562	100	

The most commonly cited reasons for screening participation were preventing sickness (56.1%, CI 53.2–59.0%), maintaining health (51%, CI 48–53.9%), and free program provision (30.9%, CI 28.2–33.6%; Table 4). Females were more likely to cite sickness prevention [OR 2.10 (CI 1.46–3.00), *p* < 0.001], where as those aged 45–54 years [OR 0.45 (CI 0.23–0.90), *p* < 0.024] or >75 years [OR 0.46 (CI 0.24–0.91), *p* < 0.025] and widows [OR 0.51 (CI 0.34–0.78), *p* < 0.002] were less likely to choose “good for my health” as a reason for screening. The screening program being free was most likely to be cited by females [OR 1.75 (CI 1.22–2.51), *p* < 0.003] and those aged 45–54 years [OR 2.55 (CI 1.21–5.40), *p* < 0.014] and 55–64 years [OR 2.86 (CI 1.39–5.88), *p* < 0.004], respectively. Conversely, those on ≥$150,001 incomes were less likely to cite this as a reason [OR 0.26 (CI 0.10–0.65), *p* < 0.004].

**Table 4:** Reasons for participation in cancer screening (those that currently participate, only).

Reasons for screening^a^	*n*	%	95% CI
Helps to prevent me getting sick	626	56.1	53.2–59.0
It is good for my health	569	51	48.0–53.9
It is free	345	30.9	28.2–33.6
Family history of cancer	234	20.9	18.7–23.4
Other	88	7.9	6.4–9.6
Do not know	15	1.4	0.8–2.3
Not stated	32	2	1.4–2.9

*^a^Participants could choose more than one answer*.

Of those who did not currently participate in screening (*n* = 445), 34.6% (CI 30.3–39.1%) stated that there was nothing that discouraged them from participation, with 55 to 64 year olds [OR 0.24 (CI 0.07–0.74), *p* < 0.04] and related/unrelated adults living together [OR 4.18 (CI 1.11–15.78), *p* < 0.035] being the least likely to cite this reason. Twenty-one percent (CI 17.2–24.8%) of non-screening respondents thought they did not need screening, while a smaller proportion stated not having time (6.9%, CI 4.9–9.7%) and the costs associated with screening (5.2%, CI 3.5–7.7%) as reasons for non-participation (Table 5).

**Table 5:** Factors that discourage respondents from participating in cancer screening (those that do not currently participate, only).

Reasons for not screening^a^	*n*	%	95% CI
Nothing discourages me	154	34.6	30.3–39.1
Do not think I need it	92	20.8	17.2–24.8
Do not have time	31	6.9	4.9–9.7
Cost	23	5.2	3.5–7.7
Location	13	3	1.8–5.0
Do not believe in it	1	0.1	^b^
Other reason	81	18.2	14.9–22.1
Not applicable	71	16	12.9–19.7
Do not know	61	13.7	10.8–17.2
Not stated	13	2.8	1.6–4.8

*^a^Participants could choose more than one answer*.

*^b^Insufficient numbers*.

The acceptability of providing different types of cancer screenings on the same day at the same location (i.e., a ‘One Stop’ screening shop) was demonstrated, with the majority of participants (85.3%, CI 81.9–88.2%) stating they would support such a program (Table 6). Interestingly, those aged ≥75 years [OR 3.78 (CI 1.67–8.57), *p* < 0.001] and those earning an income of $60,001–$100,000 [OR 2.34 (CI 1.15–4.74), *p* < 0.018] were more likely to state that they would not support or did not know whether they would support such a program. However, all other variables, such as sex, ages 30–74 years, all other income brackets, and family structure were not significant, demonstrating that a variety of people supported this concept.

**Table 6:** Proportion of respondents who would support different types of screening being offered on the same day at the same location.

Response	*n*	%	95% CI
Yes	1,332	85.3	83.4–86.9
No	15	1	0.6–1.6
Not stated	20	1.3	0.8–2.0
Do not know	195	12.5	10.9–14.2

Total	1,562	100	

## Discussion

This study had an almost even representation of male and female participants (48 and 52%, respectively) with the mean age of 54.1 years being within the current screening target population range. There was also a diversity of respondents in terms of educational level, marriage status, family structure, and income brackets. In terms of screening behavior, a number of outcomes in this study align with previous research, especially in relation to females being more engaged with screening. In general, it has been demonstrated that females use health services more than men (23), with a meta-analysis by Clark et al. also demonstrating that uptake of colorectal screening *via* fecal immunochemical testing was significantly greater in females in comparison to males, regardless of study design, screening organization or setting (24).

The most common response as to why people participate in screening, that of preventing sickness and/or maintaining health, can be considered as a motivator to screen, in terms of a form of self-care (25) or as a way of maintaining control over one’s health (26). In contrast to this, 20.8% of respondents that did not currently participate cited that they did not think they needed screening. From this particular study it is difficult to determine whether this is related to participants’ knowledge regarding screening and it is potential benefits, the individual’s own risk perception (of cancer) or a sense of ambivalence, which has previously been shown to negatively influence screening uptake (25, 27).

It is interesting to note that in this study educational level had no significant impact upon whether respondents screened or not, nor did it have an impact on the reasons provided in relation to this. In addition, income level also had minimal influence. This is contrary to the established graded association between socioeconomic status (SES), for which education and income level are a significant component, and cancer screening participation (28). This association, namely the higher the SES the higher the participation rate with the opposite being true for low SES has been demonstrated in all cancer screening modalities, including bowel (29, 30), prostate (31, 32), cervical (33), and breast (34).

As it is recognized that certain sociodemographic characteristics of individuals, such as sex, age, and ethnicity cannot be changed to improve health service use (35), attention must be directed to those factors that can be modified in relation to cancer screening utilization. As proposed in this study, one approach could be the provision of a combined health screening program (undertaken on the same day and at the same location). This type of program would target recognized structural barriers to screening, such as time, distance, and out-of-pocket costs, the reduction of which have been shown to facilitate access to and participation in organized cancer screening (11, 36).

However, who these strategies would most benefit is not entirely clear, as the review by Sabatino et al. (11) found that the evidence for reducing structural barriers to improve screening participation was stronger for breast and fecal occult blood testing in comparison to cervical screening (35), while Brouwers et al. concurred that the evidence was strong for breast screening but also for cervical cancer and in contrast to the Sabatino et al. (11) review, not for colorectal cancer screening (37). Despite these uncertainties, what is known is that there are synergies that can be potentially harnessed with the provision of an organized, integrated service such as the ‘One Stop’ screening shop.

In particular, it has been demonstrated that the decision to participate in one form of cancer screening or periodic health assessment can positively influence the decision to participate in other forms of cancer screening, including breast and cervical (38, 39) and the completion of colorectal, prostate (PSA) and mammography screening (40). A decision made potentially easier if screening services were available to the person on the same day and at the same location.

The acceptability and satisfaction of the end user to programs that combine two or more assessments and/or screening tests at the same time has been previously demonstrated, including the provision of multiple health tests on the same day to rural men (41) a community health day that provided education and basic cancer screening tests to underserved Hawaiians (42) and combined colon and endometrial cancer screening for women with Lynch syndrome (43). The consistently high acceptability and satisfaction in relation to combined testing approaches is also mirrored in this study, with 85.3% of respondents stating they would support a combined cancer screening program. However, this is the first study to demonstrate the potential acceptability of combined cancer screening being provided at the same time and location and demonstrates that this type of approach is a potentially viable and acceptable option for circumventing some of the known barriers to screening participation.

### Limitations

The predominantly Australian respondents, with a small number from different culturally and linguistically diverse backgrounds makes it likely that these groups are underrepresented in the study outcomes. This limitation is somewhat mitigated by the large sample size of this study and the other diverse sociodemographic backgrounds (in terms of age, family structure and income, and educational level) of respondents.

In addition, participants’ access/distance to health and cancer screening services was not specifically measured in this study. However, given the current context of participants having to attend multiple screening appointments at different times and locations (which inherently involves traveling), one could assume that the majority (85.3%) found the concept of a ‘One Stop’ shop a more appealing option.

## Ethics Statement

This study was carried out in accordance with the recommendations of the National Statement on Ethical Conduct in Human Research (National Health and Research Council, Australia) with written informed consent from all subjects. All subjects gave written informed consent in accordance with the Declaration of Helsinki. The NWAHS protocol was approved by the Human Research Ethics Committees of the Queen Elizabeth and Lyell McEwin Hospitals (Adelaide, SA, South Australia).

## Author Contributions

AB: manuscript preparation. KP and AT: chief investigator of the North Western Adelaide Health Study and proof reading. TG: data analysis and proof reading.

## Conflict of Interest Statement

The authors declare that the research was conducted in the absence of any commercial or financial relationships that could be construed as a potential conflict of interest.
